# Insight into Anomaly Detection and Prediction and Mobile Network Security Enhancement Leveraging K-Means Clustering on Call Detail Records

**DOI:** 10.3390/s24061716

**Published:** 2024-03-07

**Authors:** Zagroz Aziz, Robert Bestak

**Affiliations:** Faculty of Electrical Engineering, Czech Technical University in Prague, 16607 Prague, Czech Republic; robert.bestak@fel.cvut.cz

**Keywords:** call detail record, mobile networks, K-means clustering, network security, network anomaly

## Abstract

The dynamic and evolving nature of mobile networks necessitates a proactive approach to security, one that goes beyond traditional methods and embraces innovative strategies such as anomaly detection and prediction. This study delves into the realm of mobile network security and reliability enhancement through the lens of anomaly detection and prediction, leveraging K-means clustering on call detail records (CDRs). By analyzing CDRs, which encapsulate comprehensive information about call activities, messaging, and data usage, this research aimed to unveil hidden patterns indicative of anomalous behavior within mobile networks and security breaches. We utilized 14 million one-year CDR records. The mobile network used had deployed the latest network generation, 5G, with various sources of network elements. Through a systematic analysis of historical CDR data, this study offers insights into the underlying trends and anomalies prevalent in mobile network traffic. Furthermore, by harnessing the predictive capabilities of the K-means algorithm, the proposed framework facilitates the anticipation of future anomalies based on learned patterns, thereby enhancing proactive security measures. The findings of this research can contribute to the advancement of mobile network security by providing a deeper understanding of anomalous behavior and effective prediction mechanisms. The utilization of K-means clustering on CDR data offers a scalable and efficient approach to anomaly detection, with 96% accuracy, making it well suited for network reliability and security applications in large-scale mobile networks for 5G networks and beyond.

## 1. Introduction

**Figure 1:**
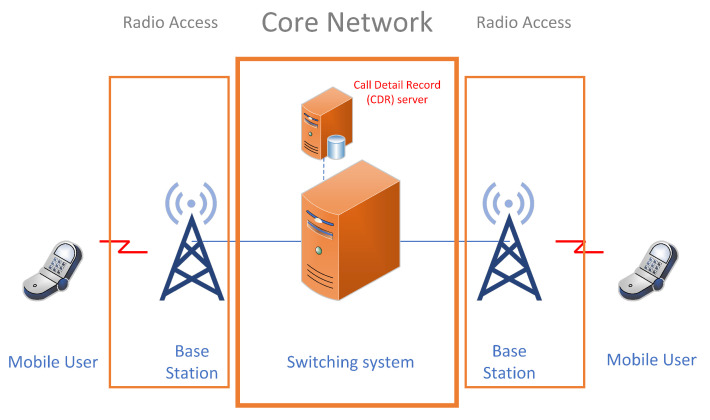
A simplified mobile network architecture with CDR database server. CDR data are a crucial component of telecommunications networks, recorded and stored in the CDR database server located at the core of mobile network and providing valuable information about voice and data communications between network users. CDR data record detailed information about each call or communication session, including its duration, timestamp, parties involved, and other relevant metadata.

In the era of pervasive mobile communication, the proliferation of smartphones and the exponential growth in mobile usage from various sources have led to a tremendous increase in the complexity and scale of mobile network infrastructures. Mobile networks, also known as cellular networks, are telecommunications networks that allow various devices to communicate wirelessly with each other and with the broader telecommunications infrastructure [1]. These networks have revolutionized communication by enabling individuals to stay connected while on the move, accessing voice, data, and multimedia services seamlessly. Mobile network operators (MNOs) face numerous challenges in ensuring the seamless operation and security of their networks amidst this dynamic landscape. One critical aspect of network management and security is the analysis of CDRs, which contain valuable information about mobile subscribers’ activities, such as number of calls, call duration, locations, data usage, date, and time. CDR data are a vital source of information for telecommunications operators (see Figure 1, providing detailed insights into call and data communication activities within their networks. These data are instrumental in various aspects of network management, including billing, network optimization, fraud detection, and customer experience management [2].

There are innumerable use cases of CDR data:

- Billing and Revenue Assurance: CDR data are used for billing purposes, ensuring that subscribers are accurately billed for their usage. This helps telecommunications operators generate invoices, track usage patterns, and reconcile billing discrepancies [3].
- Network Optimization: CDR data are invaluable for optimizing telecommunications networks. Analysis of CDR data can help identify areas of network congestion, optimize routing paths, and improve overall network performance, to enhance the quality of service for subscribers.
- Fraud Detection and Prevention: CDR data analysis is instrumental in detecting and preventing telecommunications fraud. By analyzing usage patterns and detecting anomalies in call behavior, operators can identify fraudulent activities such as call spoofing, SIM box fraud, and premium rate service fraud [4].
- Customer Experience Management: CDR data analysis enables telecommunications operators to better understand customer behavior and preferences. By analyzing call patterns, service usage, and network performance metrics, operators can tailor their services to meet customer needs and improve overall customer satisfaction.

Detecting anomalies in mobile network CDR data has become paramount for MNOs, to identify and mitigate potential security threats, fraudulent activities, and network abnormalities. Traditional methods of anomaly detection often fall short in handling the sheer volume and complexity of CDR data, necessitating the adoption of advanced analytical techniques. Among these techniques, clustering algorithms, particularly K-means clustering, have emerged as powerful tools for identifying patterns and anomalies within large datasets.

K-means clustering is a widely used unsupervised machine learning algorithm that partitions data points into distinct clusters based on their similarities [5]. However, there has been negligible prior investigation into anomaly detection, specifically in the context of utilizing CDR data for predicting and forecasting anomalies to enhance mobile network security. This paper aims to fill this gap and explore the application of K-means clustering for anomaly detection and security enhancement in mobile networks using CDR data. By iteratively optimizing cluster centroids to minimize intra-cluster variance, K-means effectively groups together CDR data points exhibiting similar characteristics. Leveraging K-means clustering in the context of mobile network CDR data enables the identification of anomalous patterns indicative of suspicious activities or network irregularities and predictive analytics.

The contributions of this study also include the following:

1. The ultimate goal of this contribution is to enhance security measures within mobile networks. By effectively utilizing CDR data and implementing K-means clustering for malicious activities, the system can identify and mitigate security threats in real time. This contributes to improving the overall security posture of the mobile network, ensuring the integrity, confidentiality, and availability of network services.
2. This research considered historical annotated incidents encountered within the network on a daily basis alongside suspicious activities identified by the algorithm. Then, these datasets were subsequently utilized to train the algorithm for future prediction. The annotations provided by the operator enhanced the accuracy of the prediction along with the historical data.
3. Prediction of anomalies: Beyond merely detecting anomalies, we explored the predictive capabilities of K-means clustering in anticipating future anomalous behaviors in mobile network traffic. By analyzing historical CDR data and identifying emerging clusters indicative of anomalous patterns, proactive measures can be taken to preemptively address security vulnerabilities and network disruptions.

Through empirical analysis and case studies, we demonstrate the efficacy of K-means clustering in detecting anomalies and enhancing security in mobile networks. By harnessing the inherent structure within CDR data, MNOs can gain valuable insights into network behavior, mitigate security risks, and ensure the resilience of their mobile infrastructure in the face of evolving threats.

In the subsequent sections of this paper, we delve into the methodology of applying K-means clustering to mobile network CDR data, discuss relevant case studies and experiments, and present practical implications for MNOs seeking to strengthen their network security posture.

## 2. Literature Review

Research into mobile network security has emphasized leveraging CDR data for anomaly detection, prediction, and enhancing security measures. Various studies have explored techniques such as machine learning, data analytics, and anomaly detection algorithms to analyze CDR data and identify potential security threats, irregularities, and vulnerabilities in mobile networks. By analyzing call patterns, traffic flows, and user behaviors, researchers aimed to develop effective security measures to safeguard mobile networks against unauthorized access, fraud, and other malicious activities [6]. These efforts have contributed to improving the overall security and reliability of mobile network infrastructures, ensuring the uninterrupted delivery of services to users, while mitigating potential risks and vulnerabilities.

Anomaly detection, prediction, and security enhancement in mobile networks are critical research areas, especially leveraging CDR data. Several studies have explored various techniques and methodologies for analyzing CDR data and addressing security challenges in mobile networks [7].

One approach involves the use of machine learning algorithms such as support vector machines (SVM) [8], random forest [9], and neural networks for anomaly detection in CDR data. These algorithms analyze call patterns, traffic flows, and user behaviors to identify anomalous activities indicative of security threats or network irregularities. By detecting deviations from normal behavior, these techniques enable proactive security measures to be deployed, thereby enhancing the overall security posture of mobile networks [10].

Another approach focuses on predictive modeling techniques to anticipate network anomalies and security breaches based on historical CDR data. By employing time-series analysis, clustering algorithms, and regression models, researchers predict potential security incidents such as network congestion, denial-of-service (DoS) attacks, and unauthorized access attempts [11]. These predictive models enable mobile network operators to implement preemptive measures to mitigate against risks and ensure uninterrupted service delivery to users.

Furthermore, research efforts have been directed towards developing dynamic security enhancement frameworks for mobile networks using CDR data analysis [12,13]. These frameworks continuously monitor call activities, analyze traffic patterns, and detect suspicious behaviors in real time. By dynamically adjusting security measures based on evolving threats and vulnerabilities, these frameworks effectively safeguard mobile networks against various security threats, including fraud, malware, and cyber attacks [14].

Additionally, studies have investigated the integration of anomaly detection techniques with network management systems to provide comprehensive security solutions for mobile networks. By combining anomaly detection with intrusion detection systems (IDS) and security information and event management (SIEM) platforms [15], researchers aim to create robust security architectures capable of detecting, preventing, and responding to security incidents in mobile networks.

Despite the critical importance of mobile network security, a notable gap exists in research that specifically addresses the intersection of security and anomaly detection and prediction within these networks. Mobile networks, characterized by their dynamic and distributed nature, are inherently susceptible to various security threats and anomalies. These can range from malicious attacks, such as distributed denial of service (DDoS) attacks and network intrusions, to benign anomalies caused by network congestion or hardware failures. Detecting and predicting these anomalies in real time is crucial for maintaining the reliability, availability, and security of mobile network services.

In spite of the growing recognition of the importance of anomaly detection and prediction in mobile network security, current research often lacks a comprehensive approach that integrates advanced anomaly detection techniques with predictive modeling methodologies and the use of CDR data. Existing studies may focus on isolated aspects of security or anomaly detection, failing to address the complex interplay between security threats and anomalous network behavior. Moreover, the rapid evolution of mobile network technologies, including the transition to 5G networks and the proliferation of Internet of things (IoT) devices, further exacerbates the challenges associated with anomaly detection and prediction. Traditional security mechanisms and detection methods may struggle to adapt to the dynamic and heterogeneous nature of modern mobile networks, highlighting the need for innovative and adaptive approaches.

In this context, this study aimed to explore and address this gap in research on security in mobile networks by integrating advanced anomaly detection and prediction techniques, leveraging the data available within mobile networks to enhance their security posture and ensure the uninterrupted delivery of critical services. Through a multidisciplinary approach that combines expertise in cybersecurity, network engineering, and data analytics, this research seeks to contribute to the advancement of mobile network security and anomaly detection capabilities, ultimately safeguarding the integrity and reliability of mobile communications in an increasingly interconnected world.

## 3. Data Acquisition and Preprocessing

Mobile network data, originating from a diverse array of sources and devices, constitute a rich and dynamic dataset that provides invaluable insights into user behavior, network performance, and emerging trends.

**Figure 2:**
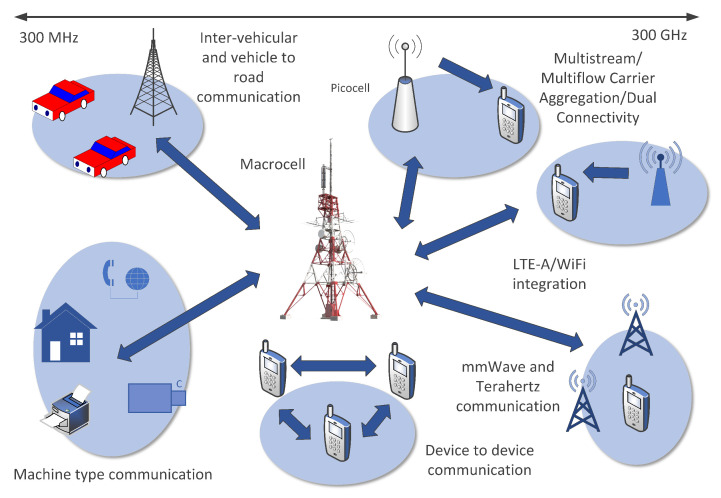
Mobile network data from various sources and devices are leveraged across diverse domains, including telecommunications, marketing, healthcare, transportation, and urban planning. These data fuel advanced analytics, machine learning algorithms, and predictive models to derive actionable insights, optimize operations, and enhance user experiences in the mobile ecosystem.

These data are generated continuously by a multitude of devices, including smartphones, tablets, IoT devices, and network infrastructure components, contributing to a complex ecosystem of interconnected data streams (see Figure 2) [16].

**Figure 3:**
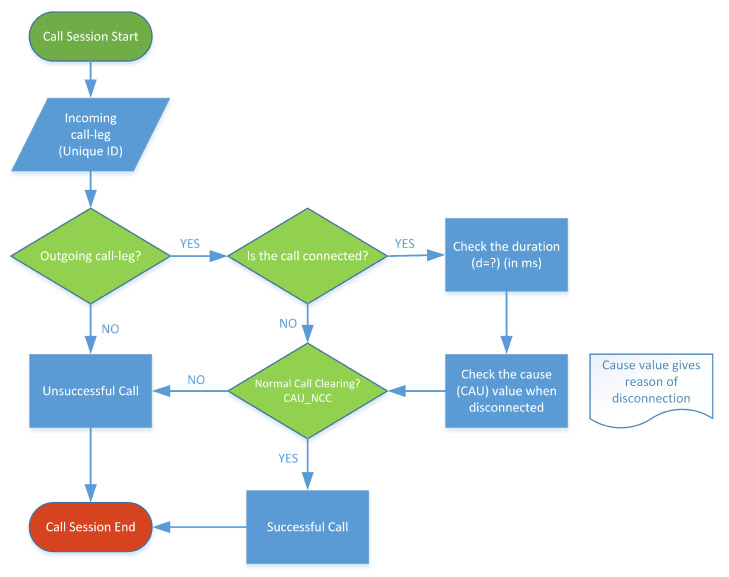
The decision tree model was utilized based on several attributes that the CDR data provide. These attributes are crucial to identifying a successful call scenario. Every call generates two call legs with unique call IDs. A successful call is disconnected once one call leg sends a disconnection command to the network after being served for a certain duration. A cause code is one of the indications revealing the status of the call disconnection.

In this study, we analyzed a dataset comprising 14 million call detail records (CDR) of successful voice calls. To isolate a successful voice call from an unsuccessful call in the CDR data, we had to employ infiltration and a decision tree model (see Figure 3).

These records were collected from a CDR database server over a period spanning from July 2016 to June 2017 and represent approximately 2 million subscribers to one of the leading cellular operators in Middle East. We utilized 10 months of the data for learning and training purposes, reserving one month for model validation.

The validation set served to fine-tune the model’s hyperparameters, prevent overfitting, and ensure accurate performance on unseen datasets. Subsequently, the test set, covering one month of the entire dataset, was employed to evaluate the final model’s performance metrics, including precision, recall, accuracy, among others. Additionally, we assessed algorithmic efficiency, including memory usage and computational time per model.

A complete CDR record for a successful call encompasses up to 100 attributes, depending on the call setup and scenarios. However, we focused on approximately 10 attributes, including call time, call date, call duration, number of calls, location services, origination leg, termination leg, calling number, and called number. These attributes are typically recorded to a flat file (text file) within the mobile core network for each call scenario before being stored in the data base. The text-based data comprise multiple lines representing the call status, relevant attributes, and information, starting with a call setup timestamp until the call is released. On the other hand, CDR files store hundreds of entire call scenario records in text-based format, with a new file created every 15 min and stored in the database.

With numerous attributes available in a single call CDR, each attribute may serve a specific purpose in analysis. For this study, we focused on leveraging the essential attributes for studying voice traffic patterns and profiles.

In our study, the process of utilizing CDR data involved several stages to prepare them for implementation in algorithms and modeling. These stages included data extraction and collection from the CDR database, profiling to assess data quality and identify key attributes, scrubbing and filtration to remove irrelevant or inconsistent data, data reduction through aggregation and dimensionality reduction, data wrangling to transform unstructured data into a structured format, enrichment with external knowledge sources, validation by dividing the data into training and testing sets, and evaluation of models to avoid overfitting and ensure unbiased estimation.

To judge the ground truth and label the CDR dataset for anomaly detection in mobile networks, we performed the following:

1. Establish a baseline of normal network behavior using historical data.
2. Consult domain experts through providing annotations to validate the baseline and identify potential anomalies.
3. Define labels that distinguish between normal and anomalous behavior through the attributes extracted from the CDR data.
4. Utilize the K-means clustering techniques to detect and predict anomalies.
5. Implement feedback mechanisms to continuously update and refine the ground truth labels based on real-time observations.

This process ensured the accuracy of anomaly detection and prediction, enhancing the security of mobile network infrastructures.

## 4. Theory and Concepts behind the Algorithm

In this section, we first discuss the K-means clustering algorithm adaptation. Then, we go through the theory and concepts of the algorithm.

### 4.1. Algorithm Adaptation

Adapting the K-means clustering algorithm for anomaly detection and prediction with CDR data involved several key modifications and considerations:

1. Feature Selection: Instead of using all available features and attributes in the CDR dataset, we carefully selected relevant attributes that captured characteristics indicative of anomalous behavior in the mobile network. This included attributes and features such as call duration, frequency of calls, geographical locations, date and time, etc.
2. Normalization: Since the features in the CDR dataset may have different scales or units, a normalization technique was added to ensure that all features contributed equally to the clustering process.
3. Distance Metric: Since we were dealing with numerical data and anomaly detection, common metrics like the Euclidean metric are not ideal for anomaly detection due to their susceptibility to biases from duplicate features or irrelevant features that do not effectively predict target attributes. We selected Mahalanobis metrics since they measure the distance of a point from the mean along each principal component in terms of standard deviations.
4. Outlier Handling: Annotations were provided by network experts for the previously reported incidents along with the historical data to enhance the identification, handling, and introduction of outlier detection mechanisms.
5. Thresholding: In anomaly detection, it is common to define a threshold to distinguish between normal (hourly/daily/weekly traffic behavior) such as peak hours, recurrent events, and anomalous clusters. We introduced thresholding techniques to identify clusters that deviated significantly from the expected behavior, indicating potential anomalies.
6. Iterative Refinement: Given the dynamic nature of mobile networks and evolving security threats, we iteratively refined our adapted algorithm based on feedback from real-world observations and ongoing monitoring of network behavior.

### 4.2. The Theory of the Algorithm

The K-means clustering algorithm is a popular method for partitioning a given dataset into K distinct, non-overlapping clusters. Mathematically, the K-means algorithm can be described as follows [17]:

- *K*: Number of clusters
- *n*: Number of data points
- *d*: Number of dimensions (features)
- $x_{i}$: Data point *i* (where i=1,2,…,n)
- $c_{k}$: Centroid of cluster *k* (where k=1,2,…,K)

Objective: The objective of K-means clustering is to minimize the within-cluster variance, also known as inertia or distortion. This is defined as the sum of squared distances between each data point and its assigned centroid within the cluster.

Mathematical Representation [18]:

1. Initialization:
  - Randomly initialize *K* centroids $c_{k}$ for each cluster.
2. Assignment Step (Expectation):
  - Assign each data point $x_{i}$ to the nearest centroid based on Euclidean distance:
    (1) $${\mathrm{argmin}}_{k}\left|\right|x_{i}-c_{k}{\left|\right|}^{2}$$
3. Update Step (Maximization):
  - Update the centroids $c_{k}$ by computing the mean of all data points assigned to cluster *k*:
    (2) $$c_{k}=\frac{1}{|S_{k}|}\sum_{x_{i}\in S_{k}}x_{i}$$
    where $S_{k}$ is the set of data points assigned to cluster *k*.
4. Repeat Steps 2 and 3 until Convergence:
  - Iterate Steps 2 and 3 until the centroids no longer change significantly or a predefined number of iterations is reached.

Objective Function: The objective function of K-means clustering is to minimize the within-cluster sum of squares (WCSS), given by

(3) $$\mathrm{WCSS}=\sum_{k=1}^{K}\sum_{x_{i}\in S_{k}}\left|\right|x_{i}-c_{k}{\left|\right|}^{2}$$

where $S_{k}$ is the set of data points assigned to cluster *k*.

Convergence Criteria: K-means clustering typically converges when one of the following conditions is met:

- The centroids do not change significantly between iterations.
- The maximum number of iterations has been reached.

The final output of K-means clustering is a set of *K* clusters, each represented by its centroid $c_{k}$, and each data point is assigned to one of the clusters based on proximity to its centroid. The algorithm aims to minimize the within-cluster variance, also known as inertia or sum of squared distances, by iteratively assigning data points to the nearest cluster centroid and updating the centroids to the mean of the data points in each cluster. We wrote K-means clustering in Algorithm 1 for our study.
**Algorithm 1** K-means clustering1:Initialize Cluster Centroids2:**for** everyiterationl **do**3:       Compute $r_{nk}$:4:       **for** for every data point $x_{n}$ **do**5:              Assign every data point to a cluster:6:              **for** every cluster *k* **do**7:                    **if** $k==\mathrm{argmin}∥x_{n}-\mu_{k}^{l-1}∥$ **then**8:                          $r_{nk}=1$9:                    **else**10:                          $r_{nk}=0$11:                    **end if**12:              **end for**13:       **end for**14:       **for** every cluster *k* **do**15:              Update cluster centroids as the mean of each cluster:16:              $\mu_{k}^{l}=\frac{\sum r_{nk}x_{n}}{\sum r_{nk}}$17:       **end for**18:**end for**

## 5. Confusion Matrix and Performance Metrics

In the realm of machine learning and classification tasks, evaluating the performance of models is crucial for assessing their effectiveness in making accurate predictions. One of the fundamental tools for assessing model performance is the confusion matrix, which provides a comprehensive overview of the model’s predictions compared to the ground truth labels.

### 5.1. Confusion Matrix

A confusion matrix is a tabular representation that summarizes the performance of a classification model. True Positive (TP), respectively True Negative (TN), which corresponds to the model correctly predicting positive instances, respectively negative instances. False Positive (FP), respectively False Negative (FN) corresponds to the model incorrectly predicts positive instances (Type I error). respectively negative instances (Type II error). Each row of the matrix corresponds to the actual class labels, while each column corresponds to the predicted class labels [19].

### 5.2. Performance Metrics

Based on the confusion matrix, several performance metrics can be derived to quantify the model’s performance across different aspects [20]:

1. Precision: Precision measures the proportion of true positive predictions among all positive predictions made by the model. It is calculated as the ratio of TP to the sum of TP and FP [21].
  (4) $$\mathrm{Precision}=\frac{\mathrm{TP}}{\mathrm{TP}+\mathrm{FP}}$$
2. Recall (Sensitivity): Recall measures the proportion of true positive predictions among all actual positive instances in the dataset. It is calculated as the ratio of TP to the sum of TP and FN [22].
  (5) $$\mathrm{Recall}=\frac{\mathrm{TP}}{\mathrm{TP}+\mathrm{FN}}$$
3. F1-Score: The F1-score is the harmonic mean of precision and recall, providing a balanced measure of a model’s performance. It is calculated as the harmonic mean of precision and recall [23].
  (6) $$F1\text{-}\mathrm{Score}=2\times \frac{\mathrm{Precision}\times \mathrm{Recall}}{\mathrm{Precision}+\mathrm{Recall}}$$
4. ROC-AUC (Receiver Operating Characteristic-Area Under the Curve): ROC-AUC measures the area under the receiver operating characteristic (ROC) curve, which represents the trade-off between true positive rate (TPR) and false positive rate (FPR) at various threshold settings [24]. It provides a comprehensive measure of a model’s ability to discriminate between positive and negative classes across different thresholds.
5. Accuracy: Accuracy measures the proportion of correct predictions (TP and TN) among all predictions made by the model. It is calculated as the ratio of the sum of TP and TN to the total number of instances in the dataset [25].
  (7) $$\mathrm{Accuracy}=\frac{\mathrm{TP}+\mathrm{TN}}{\mathrm{TP}+\mathrm{TN}+\mathrm{FP}+\mathrm{FN}}$$
6. Efficiency: Efficiency measures the computational resources required by the model to make predictions, such as the memory usage, runtime, and computational time per prediction. It is crucial for assessing the scalability and real-world applicability of the model.

## 6. Results and Discussion

**Figure 4:**
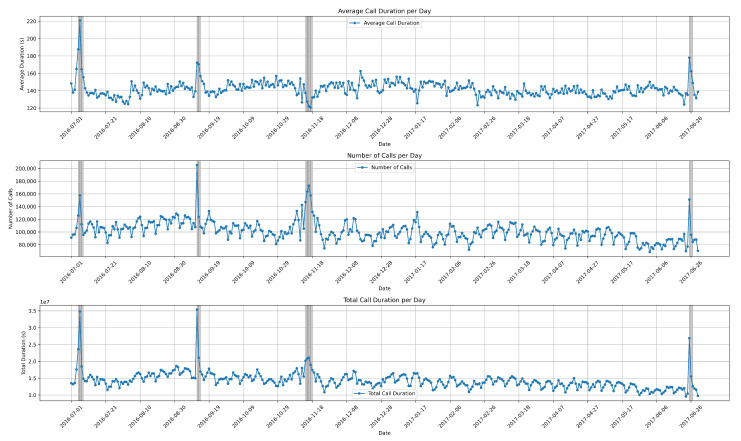
Visualization of the entire dataset, including on the y-axis the number of calls per day, the average, and total call duration per day. Date is represented on the x-axis. The grey lines are annotated anomalies.

Understanding the typical patterns of voice traffic is crucial for distinguishing abnormal behaviors. In this section, we discuss the experimental results from the dataset. We aimed to detect and predict these behaviors using K-means clustering algorithm, utilizing parameters such as date, time, number of calls, call duration, and average call duration. Initially, we visualize the entire dataset along with the attributes provided by the CDR data, as shown in Figure 4.

The first graph illustrates the average call duration per day, covering a one-year period from July 2016 to June 2017. The second graph depicts the daily number of calls throughout the entire year. Finally, the bottom graph represents the total call duration.

The data exhibited seasonality, indicating that they follow recurring patterns or variations at specific intervals, typically aligned with seasons or other fixed time periods. These seasonal fluctuations can have a significant impact on the behavior of the data, influencing trends and patterns observed over time. Understanding and analyzing seasonalized data is crucial in various fields such as economics, finance, retail, and weather forecasting.

**Figure 5:**
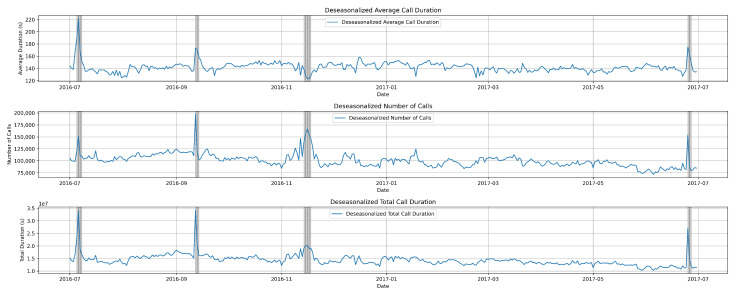
Deseasonalizing the data, which helped in reducing complexity and improving the accuracy of our results, enabling more effective anomaly detection and prediction strategies.

However, we utilized deseasonalization or seasonal adjustment on the dataset, as illustrated in Figure 5, to refine our analysis and achieve a higher accuracy.

Deseasonalization is a process used to remove the seasonal patterns or fluctuations from a time series dataset. These seasonal patterns often repeat in a regular and predictable manner over a specific period, such as daily, weekly, monthly, or yearly cycles. Deseasonalization aims to isolate the underlying trend and irregular components of the data, making it easier to analyze and interpret.

To deseasonalize our data, the following steps were followed:

1. Identification of Seasonal Patterns: Initial examination of the time series data to identify recurring seasonal patterns or fluctuations. There were certain patterns in our data, following daily, weekly, and monthly trends.
2. Estimation of Seasonal Component: Application of appropriate techniques, such as moving averages or seasonal decomposition methods, to estimate the seasonal component of the data.
3. Using Additive Model: An additive model decomposes a time series into three components: trend, seasonal, and residual (or irregular). The seasonal component is estimated by averaging the values of the data over each seasonal period (e.g., monthly averages for monthly data) and subtracting these seasonal averages from the original data to obtain the deseasonalized series. Additive model explicitly separates trend, seasonal, and irregular components, providing a clearer understanding of the underlying patterns. In addition, it can handle different types of seasonal patterns, including multiplicative ones.
4. Subtraction of Seasonal Component: Removal of the estimated seasonal component from the original data to obtain the deseasonalized data.
5. Analysis of Deseasonalized Data: Examination of the deseasonalized data to identify the underlying trend and any remaining irregular components.

Overall, deseasonalization is a critical preprocessing step in time series analysis and helps to isolate and analyze the underlying trend and irregular components in the data by removing the seasonal patterns or fluctuations. It allows for a clearer understanding of the underlying patterns in the data and facilitates more accurate analysis and modeling.

After deseasonalizing the data, we proceeded to visualize its distribution. Data distribution presents the frequency of event occurrences within specific intervals.

**Figure 6:**
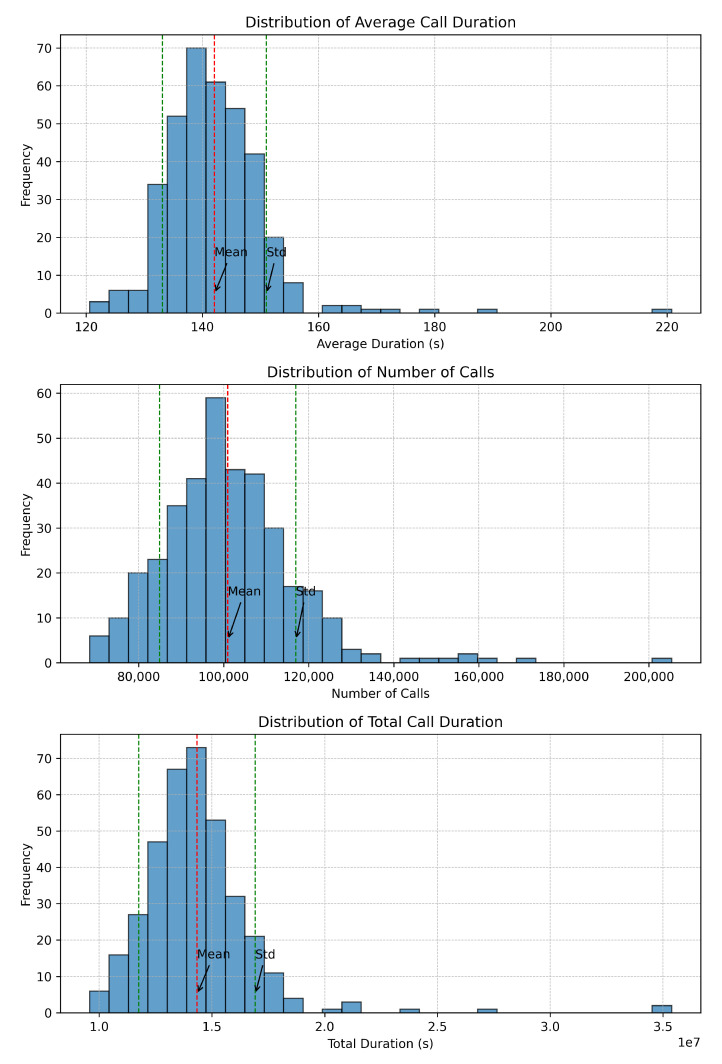
Data distribution refers to the manner in which values or events are spread out or distributed across different intervals or categories within a dataset. Understanding the distribution of data is essential in various fields such as statistics, data analysis, and machine learning, as it provides insights into the central tendency, variability, and patterns present in the data.

The distribution of data can be visualized using graphical representations such as histograms, box plots, and probability density functions. In Figure 6, we present the distributions of average call duration, number of calls, and total call duration, respectively. These visualizations can help in assessing the shape, spread, and skewness of the data distribution.

Data distribution is a fundamental aspect of understanding data and provides insights into the central tendency, variability, shape, outliers, relationships between variables, and modeling assumptions. Analyzing data distribution helps in summarizing a dataset, identifying patterns and trends, detecting outliers, and making informed decisions about data analysis and modeling techniques. In addition, it helps in understanding the spread of data values around the mean, helping to assess the stability and consistency of the data distribution.

In Figure 6, we can see the mean values for the average call duration, number of calls, and total call duration. On the y-axis, we have the frequency. On the x-axis, the distribution of data is presented. This tells us how the majority of calls along with their duration are spread over time to provide a meaningful insight into the normal traffic distribution versus outliers.

The distributions exhibit a Poisson distribution pattern, which assesses the probability of an event occurring over a period of time or distance. In Poisson distribution, events are independent of each other, and there is no restriction on the timing of their occurrence [26].

We performed experiments employing the K-means clustering algorithm for anomaly detection and prediction in the data, relying on factual annotations.

In this study, we utilized the silhouette score [27], which is a metric used to measure the goodness of a clustering technique. It quantifies how well defined the clusters are in the data. The score ranges from −1 to 1.

For each data point, the silhouette score measures how similar it is to its own cluster compared to other clusters. Higher silhouette scores indicate better defined clusters. This metric helps in selecting the optimal number of clusters for techniques like K-means clustering and evaluating the overall quality of clustering results. The obtained result of silhouette score in our study was 0.54.

The number of clusters that maximizes the silhouette score is typically chosen as the optimal number of clusters. The number of clusters was found to be five in this study.

**Figure 7:**
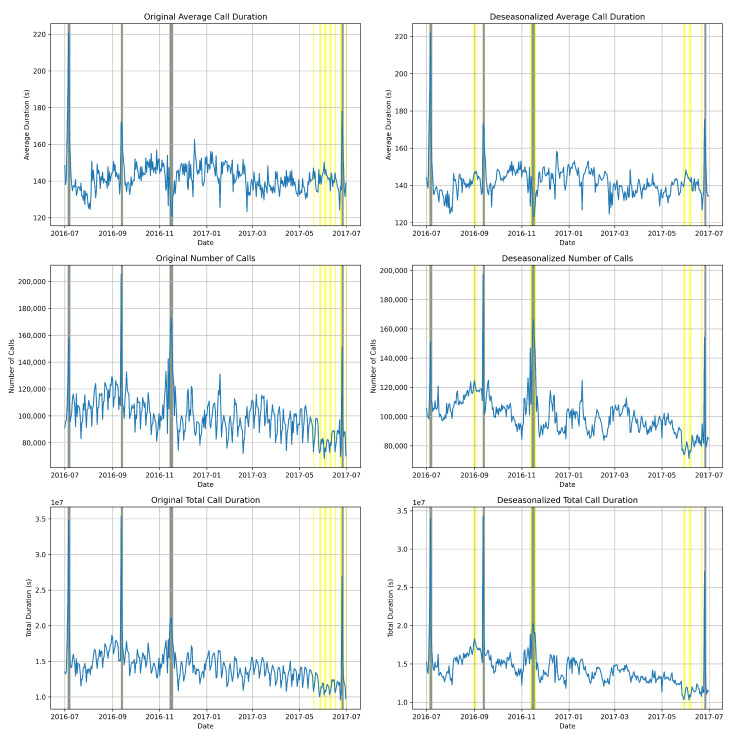
K-means clustering identifies anomalies as data points that deviate significantly from the clusters they belong to. This deviation serves as an indicator of potential anomalies based on the provided historical data.

In our analysis, we utilized both the original data (on the left) and the deseasonalized data (on the right) (in Figure 7) to provide a comprehensive comparison of accuracy. This approach enhanced graphical representation by offering additional insights into the performance of the anomaly detection system. By comparing the original and deseasonalized data, we were able to highlight any disagreements or improvements in anomaly detection across different data preprocessing techniques.

In Figure 7, the grey lines represent instances where the detected anomalies closely matched the predicted anomalies, indicating a high level of agreement between the anomaly detection system and the actual data annotations. This alignment suggests that the system effectively identified anomalies that aligned with the ground truth annotations.

Conversely, the yellow lines depict instances where the anomaly detection system predicted more anomalies than those detected by the actual data annotations. These instances may indicate areas of potential overestimation or false positives in the anomaly detection process, where the system identified anomalies that were not present in the ground truth annotations.

By visualizing both types of divergences—agreement and overestimation—we can gain a more subtle understanding of the performance of the anomaly detection system. This visual representation not only enhances the interpretability of the results but also provides valuable insights for further refinement and optimization of the anomaly detection algorithm.

**Figure 8:**
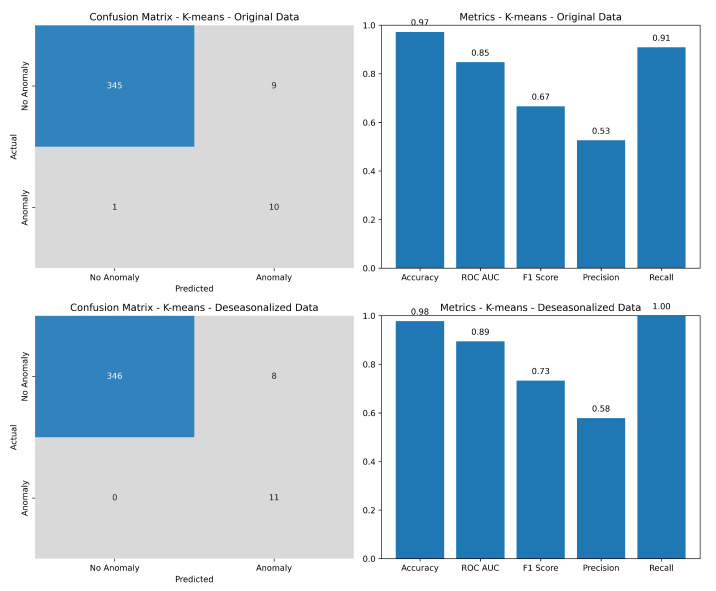
The performance evaluation showed encouraging results in terms of accuracy, ROC-AUC, and recall, especially when the data were deseasonalized. The algorithm performed very well in identifying anomalies and responded accurately to the annotated incidents.

The evaluation of the K-means algorithm’s performance, based on metric scores, is shown in Figure 8. As mentioned earlier, the metrics included precision, recall, F1-score, ROC-AUC, efficiency (numerically shown later in the factual Table 1), and accuracy. The figure represents the evaluation of both the original and deseasonalized data. In the left column, the metrics indicate better performance and higher accuracy when the data were deseasonalized.

While the performance metrics gave insights into the algorithm’s effectiveness and efficiency, the precision, recall, and F1-score did not exhibit promising results. Although the algorithm demonstrated a high detection rate for anomalies, it fell short in achieving a satisfactory F1-score, which represents the harmonic mean of precision and recall. However, the algorithm fit well in providing a high accuracy of results in detecting the annotated anomalies along with the future prediction and possibilities of additional outliers that may not be easily apparent to the factual annotations.

Table 1 demonstrates that employing the deseasonalization method yielded superior results compared to using the original data. Notably, the accuracy, as well as other performance metrics such as precision, recall, and F1-score, exhibited slight enhancements. The recall metric registered a perfect score of 1 due to the model correctly predicting all annotated outliers in the system, facilitated by deseasonalizing the data. This process mitigated the noise introduced by seasonal fluctuations, thereby smoothing the data and facilitating the identification of pertinent patterns by the model. Additionally, deseasonalizing the data improved the model fitting by eliminating seasonal effects, allowing for a focus on capturing underlying trends and patterns devoid of periodic fluctuations. Achieving a recall score of 1 is particularly desirable in scenarios where missing any relevant instance is highly undesirable, as in our case. It is worth noting that this study was the result of extensive research on the data, with network experts meticulously annotating all incidents in the network, necessitating a meticulously crafted model and dataset to achieve such exemplary performance.

**Figure 9:**
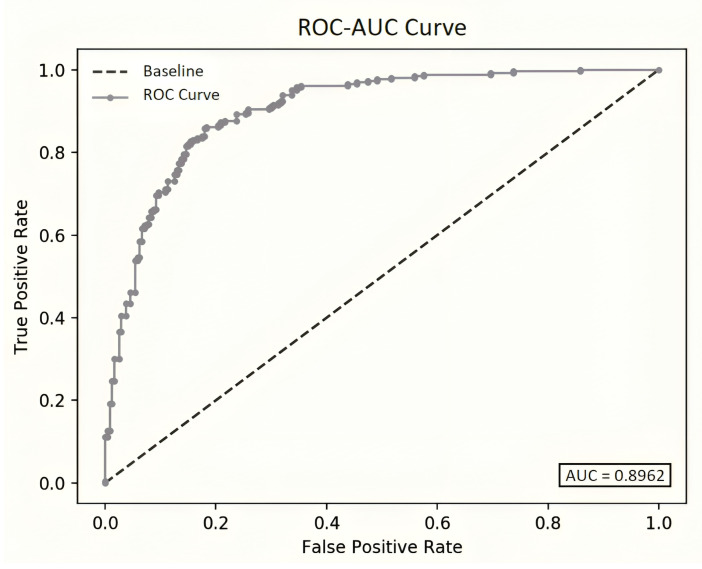
The ROC-AUC curve exhibits a notable improvement, filing 0.89 when utilizing deseasonalized data. However, this enhancement came at a slight cost in terms of increased memory usage and, consequently, model consumption time.

The ROC-AUC is visually depicted in Figure 9, illustrating the disparity between the baseline and the results obtained in our study using deseasonalized data. The baseline represents the default threshold with a straight diagonal line from (0, 0) to (1, 1), where probabilities within the range [(0.0), (0.49)] indicate negative outcomes and those within [(0.5), (1.0)] signify positive outcomes. Essentially, an AUC of 0.5 denotes random classifiers, while an AUC of 1.0 signifies perfect classifiers. The AUC serves as a concise summary of a model’s predictive ability, particularly in discerning positive outcomes when the actual outcome is certainly positive.

In general, these metrics are used to assess the performance of classification models. Depending on the specific problem and requirements, different metrics may be more important than others. For example, in scenarios where false positives are costly, precision may be prioritized, while in scenarios where false negatives are critical, the recall may be more important. Similarly, the ROC-AUC metric is useful for evaluating the discrimination capability of a model, especially when dealing with imbalanced datasets. Accuracy provides a general measure of correctness but may not be suitable for imbalanced datasets where one class dominates the other. Efficiency metrics are important in real-world applications, where computational resources are limited and faster predictions are desired.

## 7. Conclusions

This paper presents research on the characteristics of voice traffic profiles and patterns using call detail record (CDR) data. The dataset encompassed 14 million CDR records spanning one year. The study was divided into two phases. First, to comprehend anomaly and outlier behavior, understanding the normal voice traffic behavior was imperative.

We aimed to establish boundaries to distinguish daily normal traffic behavior from outliers that may occur throughout the year. These boundaries were crucial references for annotating the potential anomalies concerning the number of calls, call duration, peak hours, and daily traffic profiles. Moreover, we were aided in selecting appropriate attributes for modeling.

We visualized the entire dataset and deseasonalized it, for more accurate results. Subsequently, we illustrated the normal distribution of the available attributes, which exhibited a Poisson distribution indicating the likelihood of events occurring over time or distance. These events were assumed to be independent with no constraints on occurrence time.

The findings of our study reveal that the K-means clustering algorithm exhibited a notably high performance, achieving an accuracy rate of 96% in effectively detecting and predicting underlying anomalies. This level of accuracy held true even when applied to datasets sourced from the latest mobile network generations, including those involving 5G call detail record (CDR) data. Moreover, the performance was particularly enhanced when the dataset had undergone deseasonalization, a process aimed at removing seasonal patterns or variations from the data.

The robust performance of the K-means clustering algorithm in anomaly detection and prediction underscores its efficacy as a valuable tool in the realm of mobile network security. By successfully discerning abnormal patterns or behaviors within the network data, K-means clustering contributes significantly to bolstering the security posture of modern mobile networks, even in the context of rapidly evolving technological landscapes such as the advent of 5G networks.

These results underscore the potential of leveraging K-means clustering as a reliable method for detecting and predicting anomalies in mobile network data, thereby enabling proactive measures to mitigate security threats and ensure the integrity and resilience of mobile network infrastructures.

In our future work, we intend to introduce a comprehensive two-phase solution for anomaly detection and prediction, integrating a multi-algorithm approach. This approach will incorporate the Gaussian mixture model, mean shift, Z-score, and isolation forest algorithms.

In the initial phase, our aim is to establish a standardized methodology to isolate normal traffic behavior effectively. This phase will involve leveraging the capabilities of Gaussian mixture model and mean shift algorithms to delineate and characterize normal patterns within the data.

Subsequently, in the second phase, our focus will shift towards the detection and prediction of anomalies, coupled with enhancements in network security. Here, we will employ the Z-score and isolation forest algorithms to identify deviations from normal behavior and to predict potential anomalies within the network. Additionally, we will explore strategies for enhancing network security based on the insights gained from anomaly detection.

Overall, our proposed two-phase solution aims to provide a robust framework for anomaly detection, prediction, and network security enhancement, leveraging the strengths of multiple algorithms in tandem.

## Figures and Tables

**Table 1:** The performance metrics with the two methods used: (1) is using K-means on the original/seasonalized data, and (2) is with K-means on the deseasonalized data. We can clearly see from the numerical results that the algorithm performed better when the data were deseasonalized.

Method	Accuracy	Precision	Recall	F1-Score	ROC-AUC	Memory	Runtime [s]
1	0.961	0.421	0.727	0.533	0.85	9.25%	13.526
2	0.967	0.473	0.818	0.60	0.89	9.21%	13.511

## Data Availability

The data are from a real-world cellular operator and commercially is not applicable to share the data, it is disclosed due to security, confidentiality, and user privacy reasons. There is also a Non-Disclosure Agreement (NDA) with the cellular network operator. The contract does not allow us to share the data nor mention the name.
